# Anesthetic Management Including Postoperative Regional Anesthesia in a Young Adult Patient With Von Willebrand Disease and Osteogenesis Imperfecta

**DOI:** 10.7759/cureus.40363

**Published:** 2023-06-13

**Authors:** Dennys Rivera-Pérez, Cristian Rosa-Carrasquillo, Allan J Reyes-Sullivan, Hector Torres-Pérez, Maria J Crespo

**Affiliations:** 1 Anesthesiology, University of Puerto Rico, Medical Sciences Campus, San Juan, USA

**Keywords:** von willebrand factor, osteogenesis imperfecta, regional anesthesia, femoral nerve block, von willebrand disease

## Abstract

There are no established guidelines regarding anesthesia with a peripheral nerve block (PNB) in the young adult population with von Willebrand disease (vWD) type I. We present a case of a successful PNB outcome in a 20-year-old male patient with vWD type I, osteogenesis imperfecta (OI), and rheumatoid arthritis (RA) who underwent an intramedullary nailing surgery after suffering a left distal femur fracture secondary to a sports injury. Before the procedure, the patient was treated with HUMATE-P® [antihemophilic factor and von Willebrand factor (human)], ALPHANATE^®^ (antihemophilic factor/von Willebrand factor complex), and aminocaproic acid for hematologic control. Left femoral and popliteal nerve blocks were performed for postoperative pain control. The patient was discharged home uneventfully three days after the surgery. In this case, PNB proved to be a safe and effective alternative in the management of a vWD type I young adult patient with comorbidities. Given the lack of established guidelines, a multidisciplinary team should be involved in the pre and perioperative management of these patients due to the risk of delayed bleeding.

## Introduction

Von Willebrand Disease (vWD) is a bleeding disorder with an estimated prevalence of 1% in the US population. This condition is characterized by a quantitative reduction in von Willebrand factor (vWF) protein, which manifests as a syndrome of mucocutaneous bleeding and prolonged oozing after a surgical procedure. vWD is categorized into different subtypes based on the severity of the condition. Type I is a mild-moderate deficiency of vWF and factor VIII. Type II is described as dysfunctional vWF, and type III as a complete absence of vWF and factor VIII. In patients with this disorder, the high risk of bleeding makes perioperative management challenging, warranting a multimodal approach [1]. For perioperative management, short-term pharmacologic agents can be used as prophylaxes to prevent excessive bleeding following surgery. These agents include arginine vasopressin, factor VIII concentrates, cryoprecipitate, activated factor VII, and antifibrinolytic agents [2].

Despite the recommendations provided by the American Society of Regional Anesthesia and Pain Medicine (ASRA) and the European Society of Regional Anesthesia and Pain Medicine (ESRA) for the use of regional anesthesia in patients with a high risk of bleeding, there are currently no established guidelines for this approach in patients with vWD. Moreover, ASRA and ESRA do not define the standard of care, and state that clinical judgment should not be substituted when it comes to a specific patient scenario. To date, only a few case reports have described the use of peripheral nerve block (PNB) in adult patients with vWD. Moreover, there are no case reports in the literature describing anesthetic management in patients with concomitant vWD type I and osteogenesis imperfecta (OI). This bone disorder, also known as "brittle bone disease," is a rare connective tissue disorder that results in bone fragility and dysplasia, and affects one in 15,000-20,000 births. Patients with OI represent a challenge to anesthesiologists because this condition has been associated with abnormalities in pharyngeal anatomy, episodic apnea, and restrictive lung disease [3]. Since there are no established guidelines for the use of regional anesthesia in the postoperative anesthetic management of young adult patients with vWD and OI, this case report aims to address this issue and fill this gap in the literature.

We present the first documented case of a young adult patient with vWD type I and OI where anesthetic management was successfully achieved with PNB after prophylactic treatment with plasma-derived factor VIII concentrate that contains vWF for the prevention of bleeding.

## Case presentation

The patient was a 20-year-old male with American Society of Anesthesiologists (ASA) status 3 with type I vWD and a history of multiple fractures secondary to OI who underwent femoral intramedullary nailing. He presented to the emergency department with a complaint of pain in his left lower limb and weight intolerance after jumping while playing sports. Due to OI, he had previously received surgical management by orthopedic surgeons on various occasions. His medical history also revealed rheumatoid arthritis (RA) and seizures, and an allergy to aspirin. However, he had not received treatment for these medical conditions. A left femoral X-ray revealed a comminuted fracture coursing through the shaft and distal metaphysis, with approximately half of the shaft displaced medially. Thus, he was scheduled to undergo left intramedullary nailing. In his previous surgeries, the patient had been treated with HUMATE-P® [antihemophilic factor and von Willebrand factor (human)] and aminocaproic acid due to a history of major intraoperative bleeding.

During the pre-anesthetic evaluation, the patient’s medical history and potential complications were assessed, and the anesthesiology team chose PNB vs. spinal block due to the higher risk of catastrophic bleeding associated with the latter. The patient was offered the option of a femoral nerve block combined with a popliteal nerve block for postoperative pain control, given his history of RA and aspirin allergy. The risks and benefits of PNB and general anesthesia with a laryngeal mask airway (LMA) were discussed, with emphasis on the risks of bleeding secondary to the use of regional anesthesia. Both the patient and the legal guardian understood the risks and consented to receive the PNBs as an adjunct to general anesthesia. Due to the patient’s history of intraoperative bleeding, a hematologist was consulted by the primary care team. Intravenous (IV) HUMATE-P® (1,980 units) was administered three hours prior to the surgery, as per the hematologist’s recommendation.

**Figure 1:**
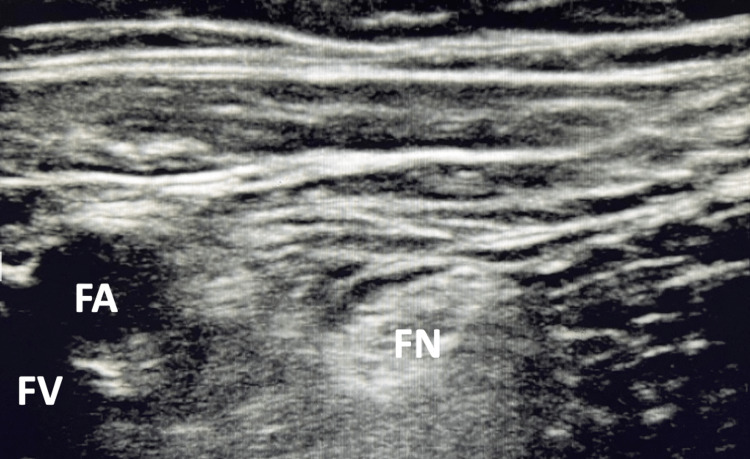
Transverse ultrasound view of the left femoral crease identifying the femoral nerve (FN), artery (FA), and vein (FV)

In the intraoperative period, the patient was placed in a supine position and monitored according to ASA standards. Premedication with midazolam (IV, 2.5 mg) was provided to the patient. General anesthesia was induced using fentanyl (IV, 100 mcg), lidocaine 2% (IV, 40 mg), and propofol (IV, 100 mg). An LMA was inserted, and general anesthesia was maintained with inhalational anesthetic. Asepsis of the left femoral crease was achieved with povidone-iodine and a sterile field was placed. The ultrasound transducer was covered and positioned over the left femoral crease to identify the femoral artery and nerve (Figure 1).

**Figure 2:**
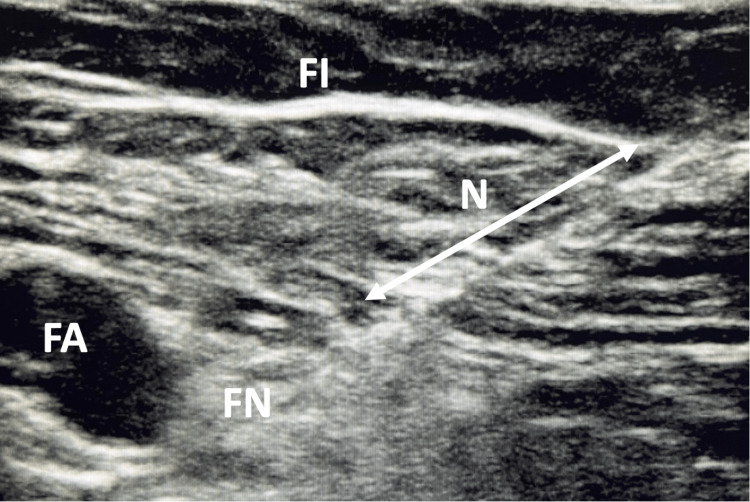
Transverse ultrasound image of the femoral nerve (FN), and artery (FA) The needle (N) was advanced medially and a single dose of 10 ml of 0.25% ropivacaine and 10 ml of lidocaine 1% with 1:200,000 epinephrine was injected between the fascia iliaca (FI) and femoral nerve (FN)

Using an in-plane technique, the needle was advanced medially toward the femoral nerve. A single dose of PNB was injected using 10 ml of 0.25% ropivacaine and 10 ml of lidocaine 1% with 1:200,000 epinephrine (Figure 2).

Adequate local anesthetic spread was observed during the procedure. Using similar aseptic and sterile techniques, the left popliteal nerve block was placed via ultrasound. After the popliteal artery and the distal sciatic nerve were identified, a single dose of PNB (10 ml of 0.25% ropivacaine and 10 ml of lidocaine 1% with 1:200,000 epinephrine) was injected using similar quantities of local anesthetic. There were no signs of bleeding, nerve injury, bone fracture, or vascular trauma throughout the procedure.

Intraoperatively, re-dosing of opioids was not required. The total surgical time was three hours, and the estimated blood loss was 200 mL. The emergence and extubation of the patient were uneventful. The patient did not complain of pain after emergence and was transferred to the post-anesthesia care unit (PACU). While in the PACU, the patient did not complain of pain and was transferred to the orthopedic ward after achieving an Aldrete score of 9. Follow-up was provided by the anesthesiology team. Nine hours after the surgery, the patient developed pain, for which the primary care team administered morphine (IV, 4 mg) as a rescue dose, and achieved resolution. On postoperative day (POD) one, the patient received an additional dose of morphine (IV, 4 mg), along with gabapentin and acetaminophen for pain management. The left femoral crease and popliteal fossa were examined by the anesthesiology team on POD one. Complete blood count (CBC) and vital signs showed no evidence of infection, and there was no bleeding. Subsequent ultrasound examination did not show hematoma formation or signs of compartment syndrome. The primary care team continued to provide medical management until the patient was discharged home three days later.

## Discussion

Orthopedic surgeries in patients with vWD carry a high risk of disabling arthropathy due to possible bleeding in the joints. Our patient not only presented with vWD type I, but also with OI, which is characterized by its predisposition to fractures, and RA, a chronic inflammatory condition that leads to painful joints. To our knowledge, this is the first report of the successful use of PNB for postoperative pain management in a patient with concomitant vWD type I, OI, and RA treated with factor VIII concentrates that contain vWF.

For postoperative pain management of patients with vWD, an important consideration is to avoid non-steroidal anti-inflammatory drugs (NSAIDs), which limits the options for pain management. Moreover, patients with OI may have multiple exposures to opioids and other analgesics over time due to prior history of surgery, rendering perioperative pain management a challenge [4]. Thus, the use of multimodal anesthetic methods for postoperative pain has become crucial for pain management in these patients. PNB as a modality for lower extremity surgery is becoming more common than neuraxial anesthesia due to lower rates of hypotension and urinary retention [5]. PNB may provide satisfactory postoperative analgesia, ensuring early rehabilitation and rapid recovery [6]. Also, the combination of PNB with general anesthesia helps reduce the number, dose, and frequency of IV drugs used intraoperatively and the time spent in the PACU [7].

Hematomas resulting from PNB tend to be less catastrophic when compared to neuraxial anesthesia because it develops in a more compliant space, which may result in less nerve impingement [8]. Hematoma formation in neuraxial anesthesia carries a high risk of permanent neurologic damage due to the catastrophic nature of bleeding into a fixed, noncompressible space. Similar risks apply to patients undergoing peri-neuraxial, deep plexus, or deep PNB due to a similar chance of bleeding [9]. Management of patients undergoing PNB, however, is case-dependent and should be based on ultrasound guidance, site compressibility, vascularity, and the coagulation status of the patient.

The data/studies in the literature regarding morbidity related to hematoma formation following PNB in coagulopathic patients are scarce, and none involve patients with vWD. Rodríguez et al. [10] reported the first case of intraneural hematoma formation following a nerve stimulation-guided femoral block in a patient with undiagnosed factor XI deficiency. The patient presented with the onset of paralysis 10 days after surgery, which warranted early nerve decompression. By contrast, the diagnosis of vWD type I in the current case was known, and the patient was treated prophylactically with HUMATE-P®, effectively achieving homeostasis during surgery. Moreover, the use of ultrasound guidance reduces the risk of vascular violation during block administration. Following femoral and popliteal PNB, no hematoma formation, infections, or signs of compartment syndrome were observed either postoperatively or in the long term.

Our management outcome was consistent with the findings of Vanarase et al. [11], who described the use of femoral and sciatic nerve block in 13 patients with hemophilia and in two patients with vWD undergoing total knee replacement surgery; none of them developed hematoma or infection at the injection site. The patients were treated with factor VIII or desmopressin (DDAVP) one hour prior to surgery. The authors concluded that the risks of hematoma formation and nerve damage are low, given that the sciatic nerve is not surrounded by large vessels and the femoral artery and vein are compressible against the femoral head.

The use of regional anesthesia in patients with concomitant OI and vWD has not been previously described. In addition to the common complications associated with high-risk surgeries, excessive bleeding stands out as a major issue in this population. To prevent excessive bleeding, multiple pharmacological approaches may be utilized perioperatively, including arginine vasopressin, factor VIII concentrates, cryoprecipitate, activated factor VII, and antifibrinolytic agents [2]. A meta-analysis involving 44 cases of patients with OI who underwent PNB reported complications in only two cases [4]. In the first case, a patient with OI type III developed a new median nerve neuropathy after the placement of a supraclavicular catheter following a distal humerus fracture. However, the true etiology of the neuropathy was unclear due to the fracture extending above the fixation point. In the second case, a parturient with worsening restrictive lung disease who underwent a cesarean section under general anesthesia was catheterized for a bilateral transversus abdominis plane block to provide postoperative analgesia. The patient remained intubated postoperatively for a short period of time, most likely due to her preoperative respiratory status, rather than for placing the catheters. In this review, the negative sequelae related to the use of PNB were insufficient to support relative contraindication in patients with OI.

The major strength of this report is that it describes a unique case of a young adult patient with multiple comorbidities successfully treated with a multimodal approach not described in the literature so far. The main limitation, however, is that the outcome in our patient may differ from that of patients in the adolescent age group who are younger than him. Although there is a theoretical benefit of using regional blocks in patients with coagulation disorders and OI, there is no conclusive evidence to make definitive recommendations yet. PNB should be considered on a case-by-case basis while accounting for inherent risks and benefits in patients with vWD type I and OI. A multidisciplinary team comprising hematologists, nurses, pharmacists, anesthesiologists, and surgeons is warranted for the perioperative management of patients with vWD and OI.

## Conclusions

The findings of our case report suggest that in patients with non-severe vWD type I and OI, the use of PNB as a modality for postoperative pain management is a suitable alternative. This not only reduces the amount of opioids administered but also helps provide analgesia in patients with limited options for pain management. In addition, the use of ultrasound guidance in this population is essential when performing the PNB to reduce the risk of vascular damage. Proper hematological management is also crucial to reduce the risk of bleeding. Furthermore, we recommend post-procedural vigilance and surveillance for signs of PNB-associated bleeding and other potential complications.
